# Supplementary material for: Cerebellar Contribution to Preparatory Activity in Motor Neocortex
Source: Neuron. 2019 Aug 7;103(3):506–519.e4. doi: 10.1016/j.neuron.2019.05.022 (PMC6693889; doi:10.1016/j.neuron.2019.05.022)
Supplement: Document S1. Figures S1–S12 [file mmc1.pdf]

Neuron, Volume *103*

Supplemental Information

Cerebellar Contribution to Preparatory Activity in Motor Neocortex

Francois P. Chabrol, Antonin Blot, and Thomas D. Mrsic-Flogel

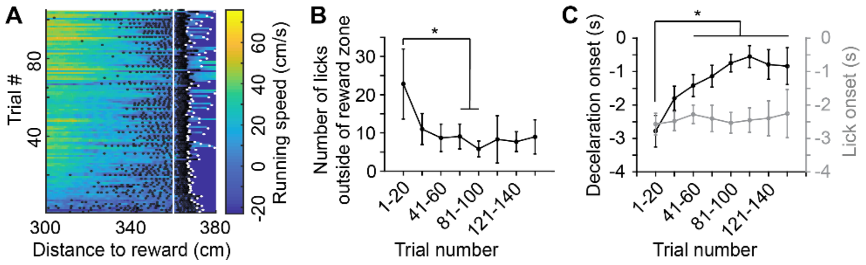

Figure S1. Refinement of motor behaviour in anticipation of reward (related to Figure 1)
(A) Summary of behavior inside the rewarded section of the virtual corridor for an example recording session of a trained mouse (including all trials). Running speed over distance is color-coded. Black and white dots represent individual lick times and end of trial, respectively. Reward position is indicated by the vertical white line.
(B) Number of false alarm licks averaged in chunks of 20 trials over the course of recording sessions across all mice (n = 21). Error bars are standard deviations. Multiple comparison test after a Kruskal-Wallis test shows significant decrease of false alarm licks over 61-80 trials.
(C) Summary plots of deceleration and lick rate onsets relative to reward time (respectively, onset of 20% decrease and increase of Z-score values) averaged in chunks of 20 trials across recording sessions. Error bars are standard deviations. Time between deceleration onset and reward delivery significantly decreased over 41-60 trials (multiple comparison test after a Kruskal-Wallis test).

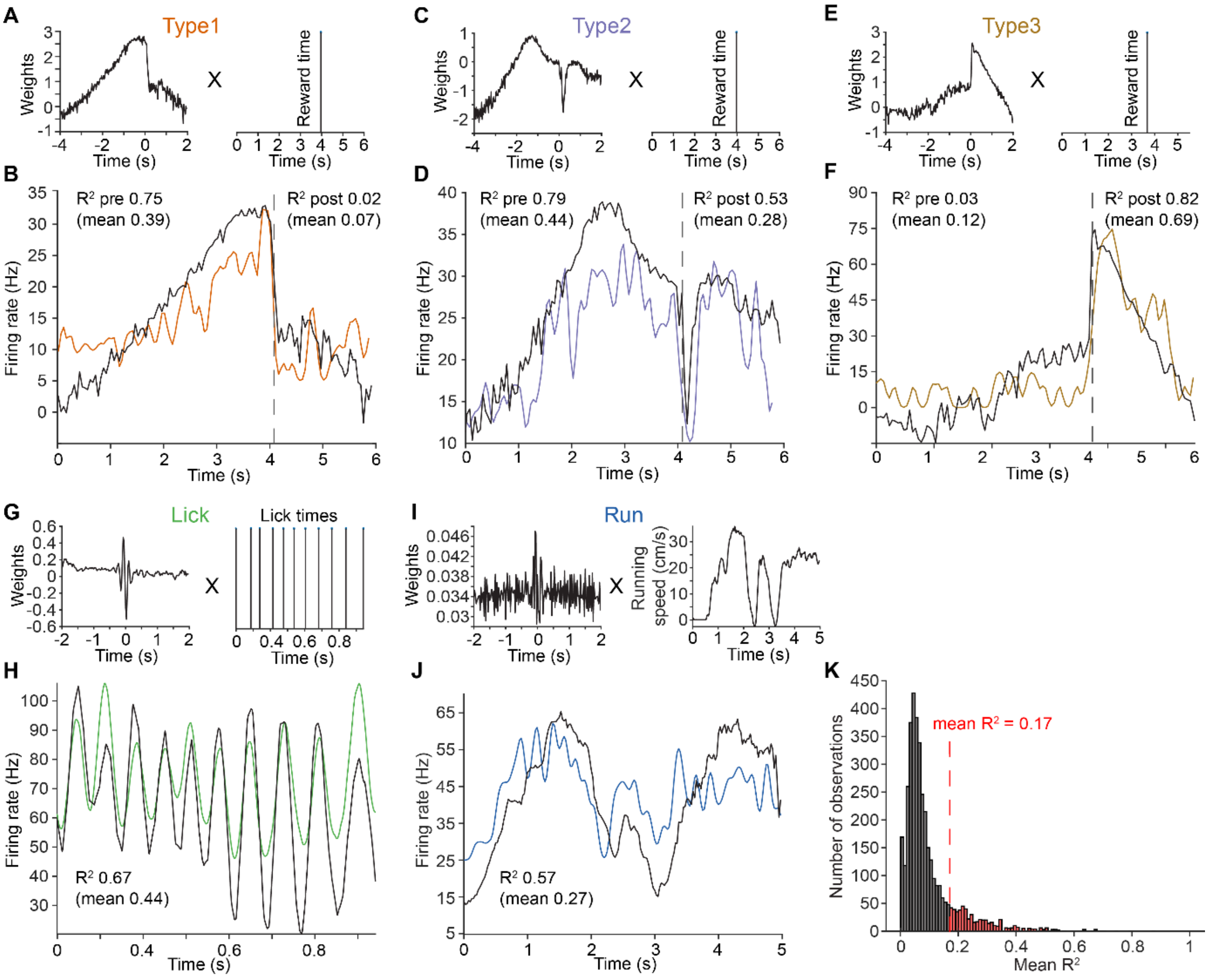

Figure S2. Unit classification using generalized linear model single-trial predictions from external covariates (related to Figures 1,2,3 and 6)

(A) The weight vector (left) resulting from linear regression of spike times and reward times for a unit classified as type 1 is convolved with single trial events (reward time, right) to model neuronal activity.

(B) The goodness of fit of GLM model (black trace) to neuronal firing rates (color-coded trace) are estimated over single trials. The coefficient of determination value for the example trial is written in the panel, the mean coefficient of determination across trials is given in parentheses. For reward times-based models the fit was evaluated over 2-time windows: -4 to 0, and 0 to 2 s around reward times, as indicated by the grey dashed line, to distinguish pre-reward only (type 1), pre and post-reward (type 2), and post-reward only (type 3) neuronal activity. Coefficient of determination values for pre- and post-reward fits are given on the left side and right side of the traces respectively (B, D, F).

(C, D) Same as (A,B) for a unit classified as type 2.

(E, F) Same as (A,B) for a unit classified as type 3.

(G, H) Same as (A,B) for a unit with activity best predicted by lick times.

(I, J) Same as (A,B) for a unit with activity best predicted by running speed.

(K) Distribution of mean coefficient of determination values between all units and GLM models obtained from each external covariate (reward times, lick times, and running speed). A threshold criterion for significant goodness of fit (mean $R^2 > 0.17$, red dashed line) was obtained by performing k-means clustering on the R^2 distribution with 2 clusters.

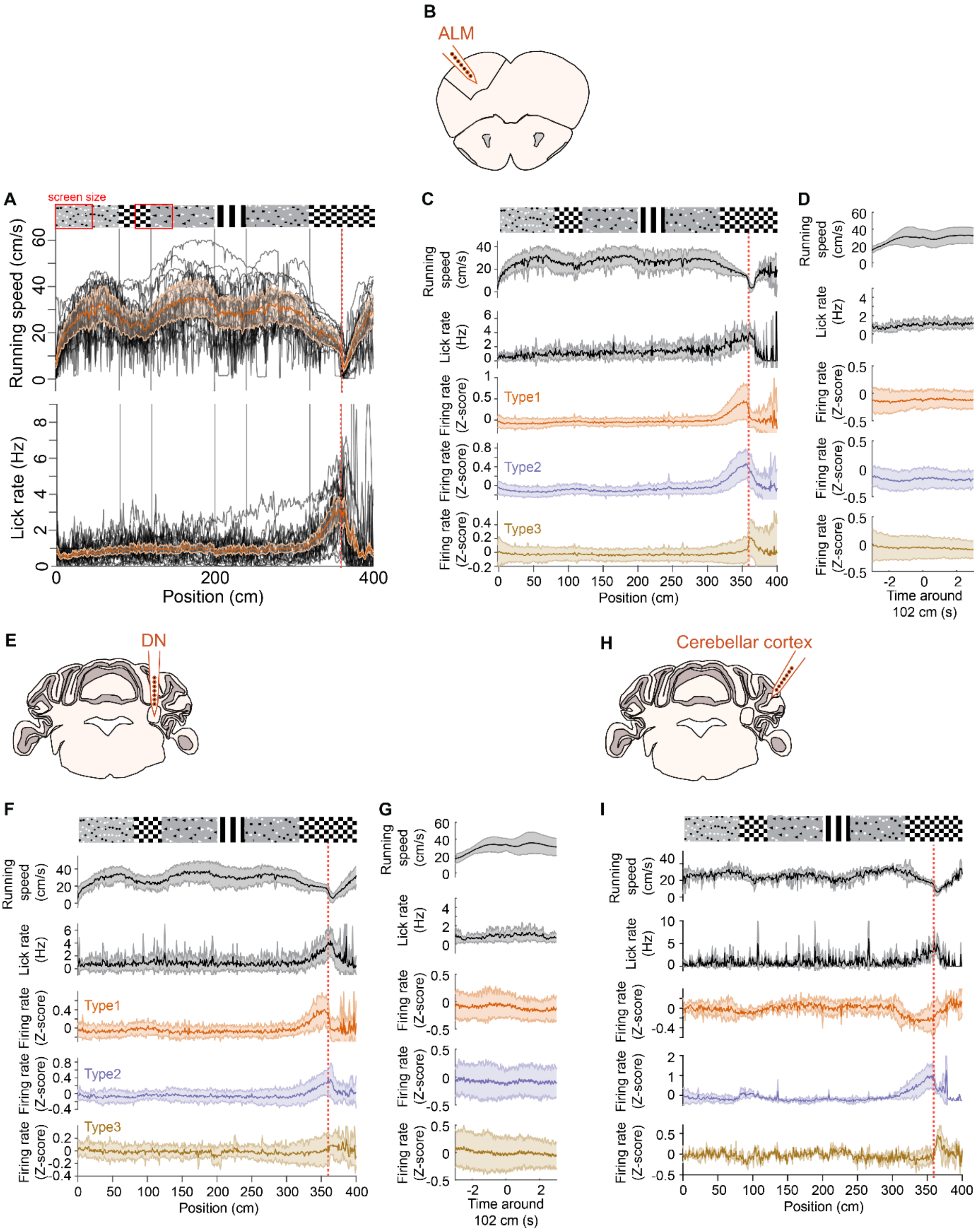

Figure S3. Type 1-3 ALM and DN neurons and lateral crus 1 Purkinje cells are selectively active around reward position (related to Figure 1, 2 and 5)

(A) Running speed (top) and lick rate (bottom) profiles for all mice (black curves, 21 expert mice) and population average (orange trace, shading is SD) as a function of position inside the virtual corridor. The reward position is indicated by the red vertical dotted line. The visual patterns displayed on the corridor walls shown above the running traces are aligned to the position at which they fully appear in the field of view of the mice (i.e. when they reach the back edge of the monitors). The full field of view of the mouse which corresponds to 47 cm (monitor width) is indicated by the red square box

around the visual patterns. Note the consistent deceleration of mice around the 1st non-rewarded checkerboard and to a lesser extent around vertical gratings. The trough of mouse speed around the 1st checkerboard occurs around 102 cm, when the mouse is halfway out of the checkerboard as indicated by the second red box.
(B,E,H) Schematic showing recording location in the anterolateral motor cortex (ALM; B), dentate nucleus (DN; (E)) and lateral crus 1 (H).
(C,F,I) Type 1-3 ALM neurons (line is mean and shaded area is SD from 37, 29, and 88 neurons respectively, recorded from 6 mice; (C)), DN neurons (20, 32, and 57 neurons respectively, recorded from 15 mice; (F)) and Purkinje cells (8, 4, and 2 neurons respectively, recorded from 3 mice; (I)) are mostly active around reward position (vertical red dotted line). Note the slight modulation in neuronal activity around the 1st non-rewarded checkboard at which position mice decelerate.
(D,G) Mean (line) and SD (shaded area) of running speed, lick rate, and type 1-3 ALM (D) or DN (G) neurons activity around the time of mice deceleration trough occurring after the appearance of the 1st checkerboard (at 102 centimetres from the start of the corridor).

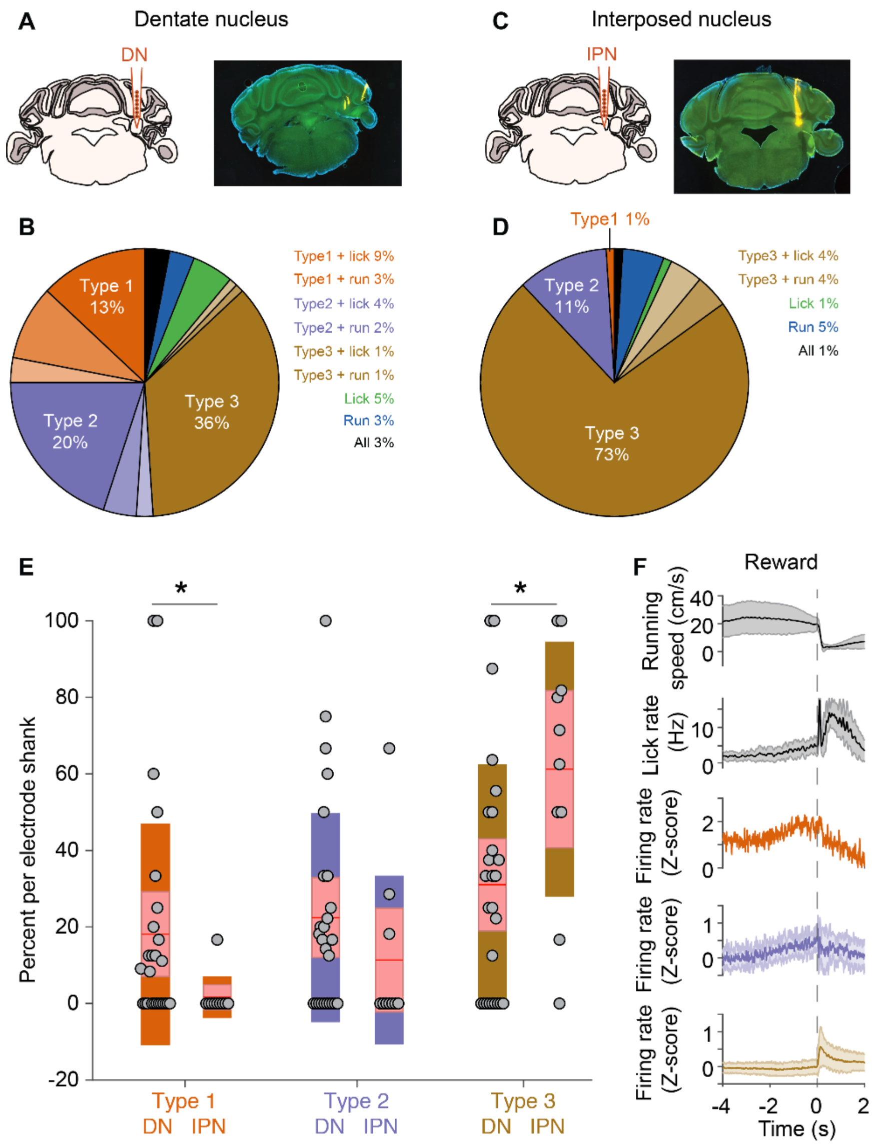

Figure S4. Preparatory activity is significantly more present in the dentate than in the interposed nucleus (related to Figure 2)

(A) Left: Schematic of recording location in the Dentate nucleus (DN). Right: Coronal brain slice from a DN recording (yellow traces are from DiI staining on recording electrode shanks, tract in lateral crus 1 is from simultaneous recording in the cerebellar cortex).

(B) Summary DN neuron classification.

(C) Same as (A) but showing recording location in the more medial interposed nucleus (IPN).

(D) Summary IPN neuron classification.

(E) Summary plot of percentage of type 1-3 neurons amongst classified units per electrode shank for DN (left columns) and IPN (right columns). Proportion of type 1 neurons is significantly lower in IPN (n = 1/74, 5 mice) than in DN (n = 20/170, 15 mice; p = 0.024, Wilcoxon Rank-Sum test). There is no significant difference in the proportion of type 2 neurons between IPN (n = 8) and DN (n = 32; p = 0.16, Wilcoxon Rank-Sum test). Proportion of type 3 neurons is significantly higher in IPN (n = 54) than in DN (n = 57; p = 0.02, Wilcoxon Rank-Sum test).

(F) From top to bottom, average (line) and SD (shaded area) running speed, lick rate, and type 1-3 IPN neuronal activity centered on reward (grey dotted line).

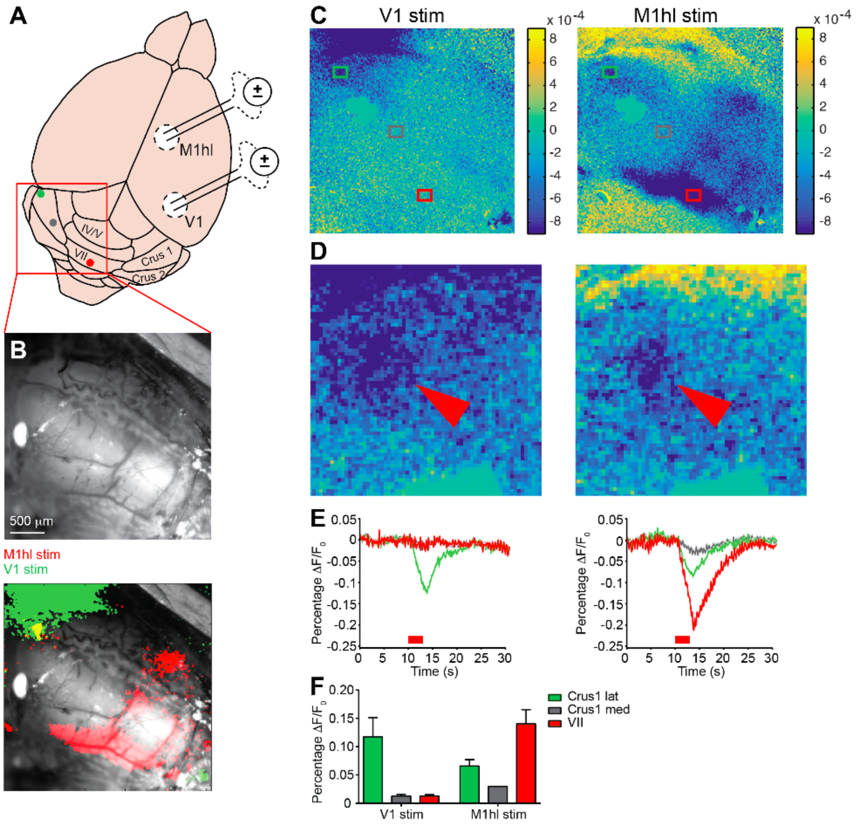

Figure S5. Mapping visuomotor cerebellum (related to Figure 3)

(A) Schematic of electrical stimulation for identification of a cerebellar region activated by inputs from primary visual cortex (V1) and hind limb-related motor cortex (M1hl). Coloured dots correspond to regions from where hemodynamic signals were measured in (C-E): lateral crus 1, medial crus 1 and lobule VII.

(B) Top: wide-field image of the cerebellar surface. Bottom: same image overlaid with 20-trials of hemodynamic signals averaged across sessions (showing only peak decrease from baseline) for electrical stimulation of V1 (green) and M1hl (red) for an example recording. Note that the large haemodynamic signal induced by V1 stimulation outside of the cerebellum probably comes from the cerebral venous sinus.

(C) Wide-field image of 20-trials average hemodynamic signals color-coded according to percentage change of infrared reflectance from baseline for V1 (left) and M1hl stimulation (right). Coloured rectangles indicate the areas from which the signals were measured from.

(D) Zoom on the lateral crus 1 zone from the wide-field image in (C). Red arrowheads indicate region of max hemodynamic response.

(E) Mean response time courses after V1 (left) and M1hl stimulation (right) color-coded according to the sampled areas in (C). ΔF/F0, normalized change in reflectance. The timing of electrical stimulation is indicated by the red bar below the traces.

(F) Summary of peak hemodynamic response values after V1 (left) and M1hl (right) stimulations for each cerebellar area (*n* = 4 mice).

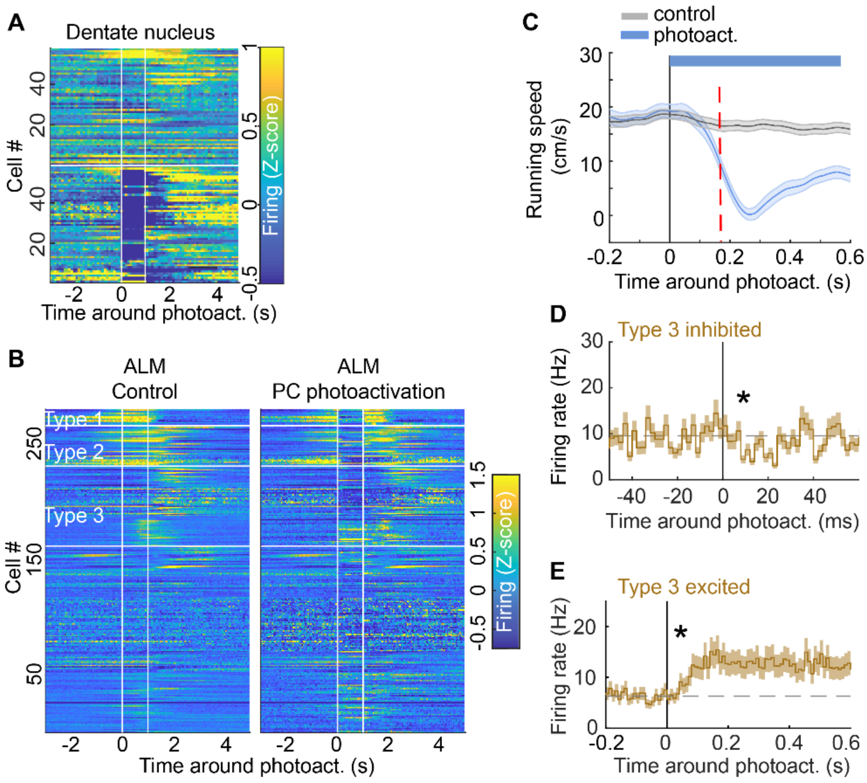

Figure S6. Effects of cerebellar Purkinje cells photoactivation on DN and ALM neuronal populations and on mouse locomotion (related to Figure 3)

(A) Purkinje cell (PC) photoactivation efficiently silenced most dentate nucleus (DN) neurons (one line per neuron). Upper half: control trials. Lower half: photoactivation trials.

(B) PC photoactivation efficiently suppressed activity in most type 1 and type 2 ALM neurons (one line per neuron). Purkinje cell photoactivation (right) compared to control trials (left). The effects of photoactivation on type 3 and unclassified ALM neurons were more diverse.

(C) PC photoactivation (‘photoact.’) induced sudden mouse deceleration. The time of significant deviation from running speed in control trials is indicated by the red dotted line at 170 milliseconds (running speed significantly lower than control speed minus one standard deviation, calculated from 10 ms bins, $p < 0.005$, Wilcoxon Rank-Sum test).

(D) Average response profiles of firing rate type 3 ALM neurons with significant reduction in activity compared to control trials (star: first significant bin at 8 milliseconds; see Methods), during the first 60 milliseconds following PC photoactivation onset (vertical line). Shaded area represents SEM.

(E) Same as in (D) with 10 ms binning for type 3 ALM neurons which activity significantly increases after PC photoactivation (star: first significant bin at 40 millisecond). Shaded area represents SEM.

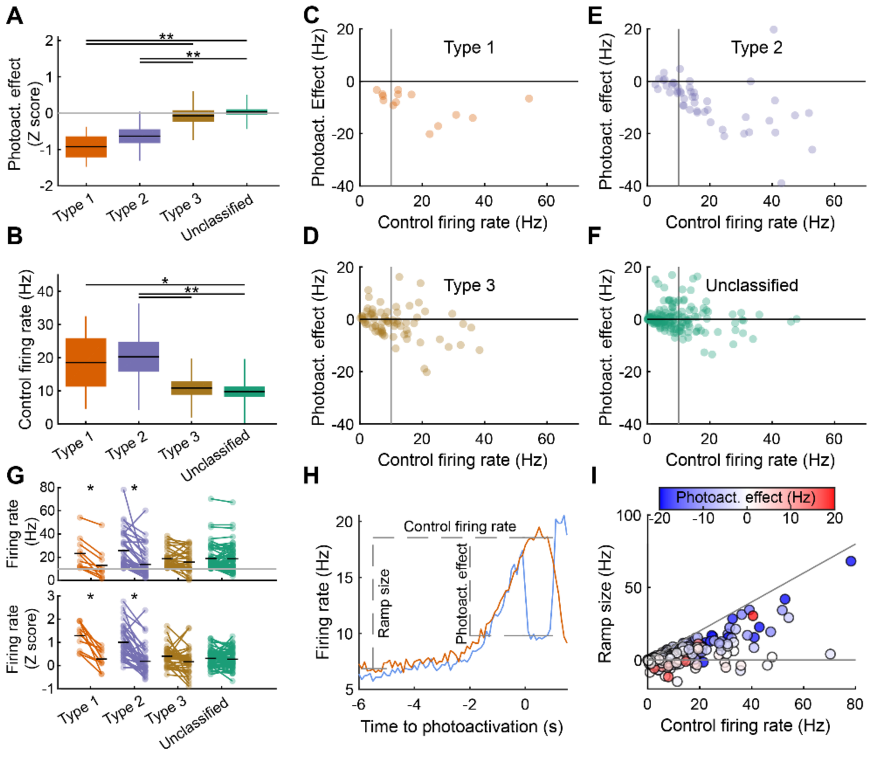

Figure S7. Photoactivation effect is not explained by firing rate (related to Figure 3)

(A) Type 1 and type 2 neurons were significantly more inhibited by photoactivation than type 3 and unclassified neurons. Box plots represent the 95 % confidence intervals and SD, black line is the median.

(B) Type 1 and type 2 neurons had higher firing rate in control trials.

(C-F) The effect of photoactivation did not seem to be explained by this difference in firing rate as type 1 (C) and type 2 neurons (E) were mostly inhibited regardless of firing rate and inhibited type 3 (D) and unclassified neurons (F) had similar firing rates to that of unmodulated neurons.

(G) When selecting only neurons firing at more than 10 Hz in control condition (vertical grey line in C-F), type 1 and type 2 neurons were significantly inhibited while the firing rate of type 3 and unclassified neurons was not significantly affected (see Methods).

(H-I) The photoactivation effect (blue trace) was correlated with the size of the ramp (orange trace) but not with the control firing rate (see Methods).

(A,B,G) * indicates $p < 0.005$, ** $p < 0.0001$ (Wilcoxon Rank-Sum test with Bonferroni correction for multiple comparisons).

(A-G) n = 14, 49, 74, 165 respectively for type 1, 2, 3 and unclassified neurons.

(I) n = 302 neurons, including all types.

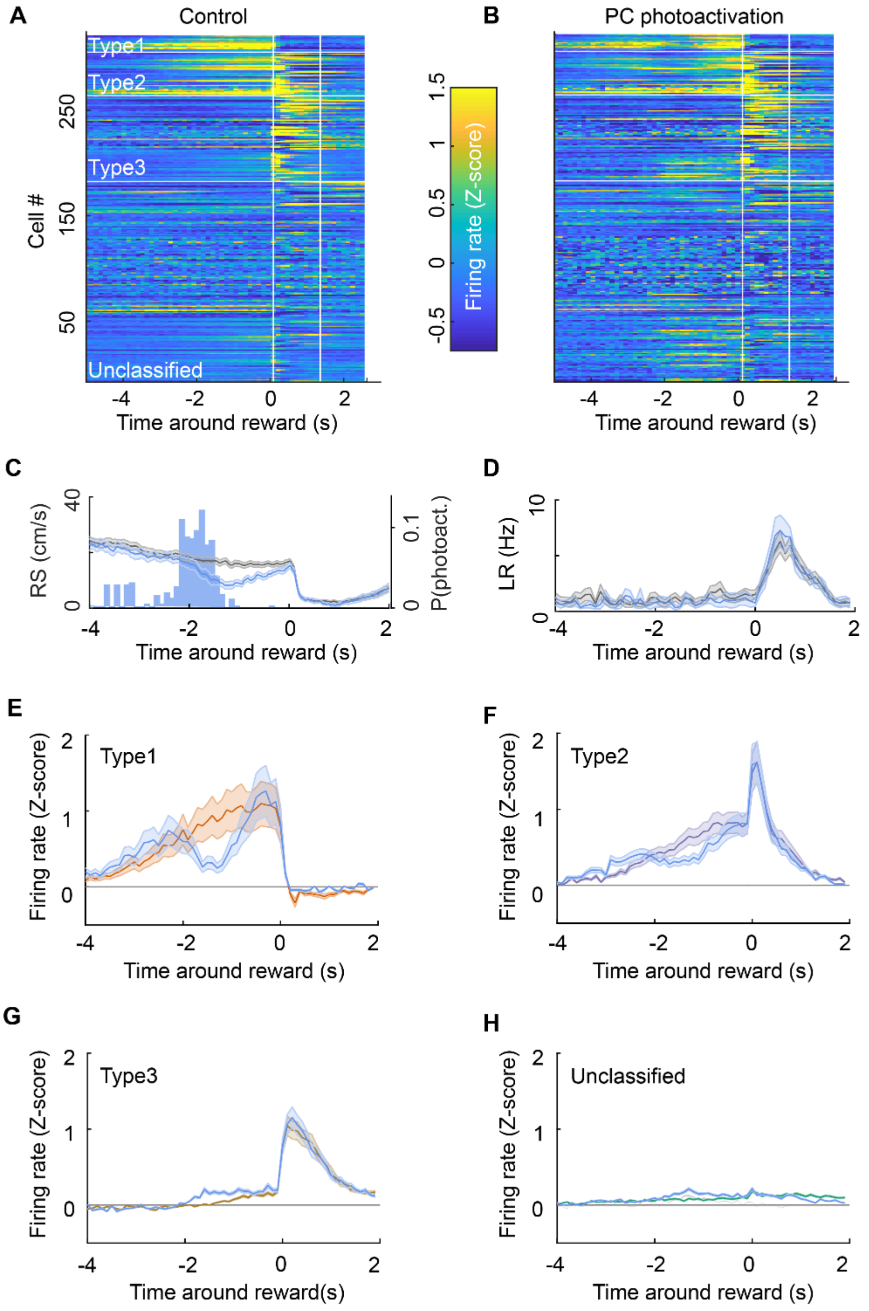

Figure S8. Effects of cerebellar photoactivation on ALM activity aligned to reward time (related to Figure 3)

(A,B) Response profiles colour-coded by firing rate (z-scored) for all ALM neurons sorted by cell type in control condition (A) and with photoactivation of Purkinje cells (PC, B) aligned on time of reward delivery. The photoactivation started 20 cm in advance of the reward position, corresponding to about 2 s before reward time (see photoactivation probability distribution in panel (C)), and lasted 1 s.

(C,D) Average profiles around reward time in photoactivation trials (blue lines) and control trials (grey lines) for running speed (RS, C) and lick rate (LR, D) and distribution of photoactivation probability (P(photoact.)) in reward trials (C**,** blue histogram). Shaded areas represent SD.

(E-H) Firing rate profiles aligned to reward time for type 1 (E), type 2 (F), type 3 (G) and unclassified ALM neurons (H) during photoactivation (blue traces) and control trials (coloured traces). Shaded areas in (C-H) represent SD.

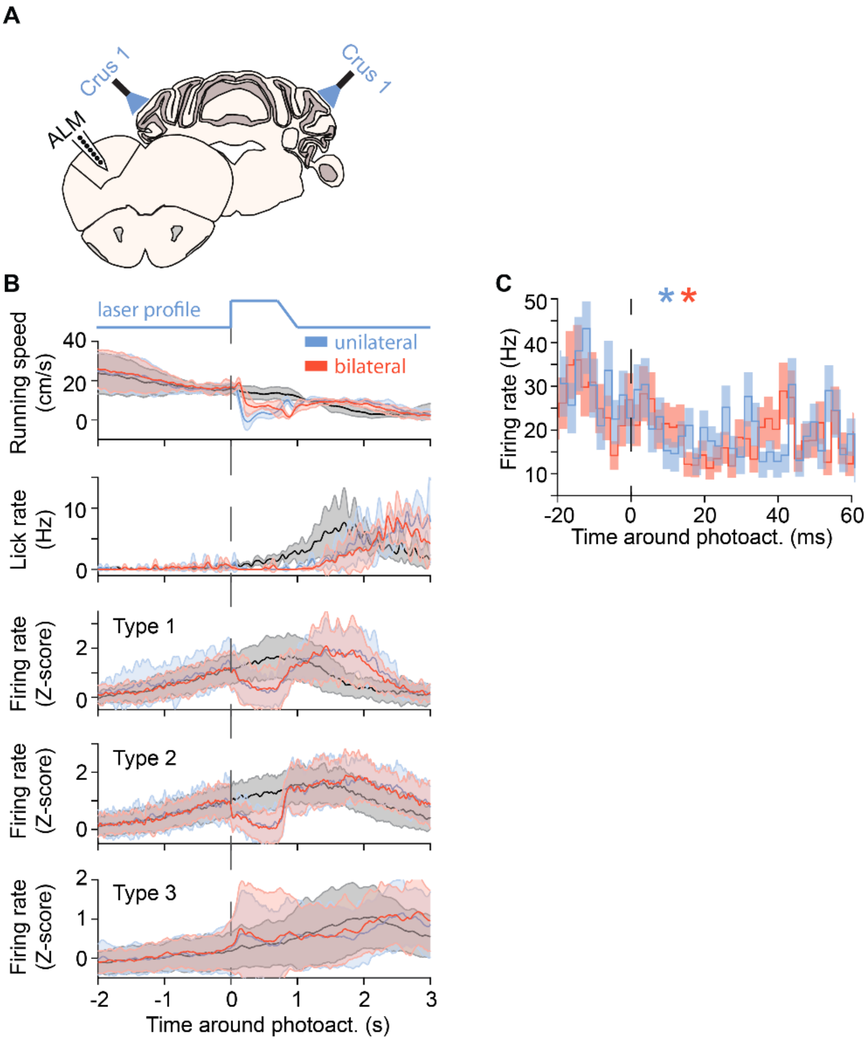

Figure S9. Comparison of the effect of unilateral and bilateral crus 1 PC photoactivation on ALM activity (related to Figure 3)

(A) Schematic of experiments. The anterolateral motor cortex (ALM) was recorded in L7-ChR2 mice preforming the task during photoactivation of Purkinje cells (PCs) in lateral crus 1 either unilaterally (only left crus 1) or bilaterally.

(B) From top to bottom, average (line) and standard deviation (shaded area) of running speed, lick rate, and type 1-3 ALM neuronal activity during control (grey), unilateral crus 1 (blue), or bilateral crus 1 PC photoactivation (red) aligned on photoactivation onset (vertical dotted line). The laser activation profile is illustrated above the running speed traces. A ramping offset was introduced to limit the rebound of neuronal activity.

(C) Average response profiles of firing rate from type 1-2 ALM neurons with significant reduction in activity compared to control trials, during the first 30 milliseconds following PC photoactivation onset (vertical dotted line) in unilateral (blue) or bilateral crus 1 photoactivation trials (red). Shaded areas represent SEM. Color-coded stars indicate first bin where firing rate significantly deviates from baseline (8 ms for unilateral crus 1 photoactivation, 14 ms for bilateral crus 1 photoactivation; see Methods).

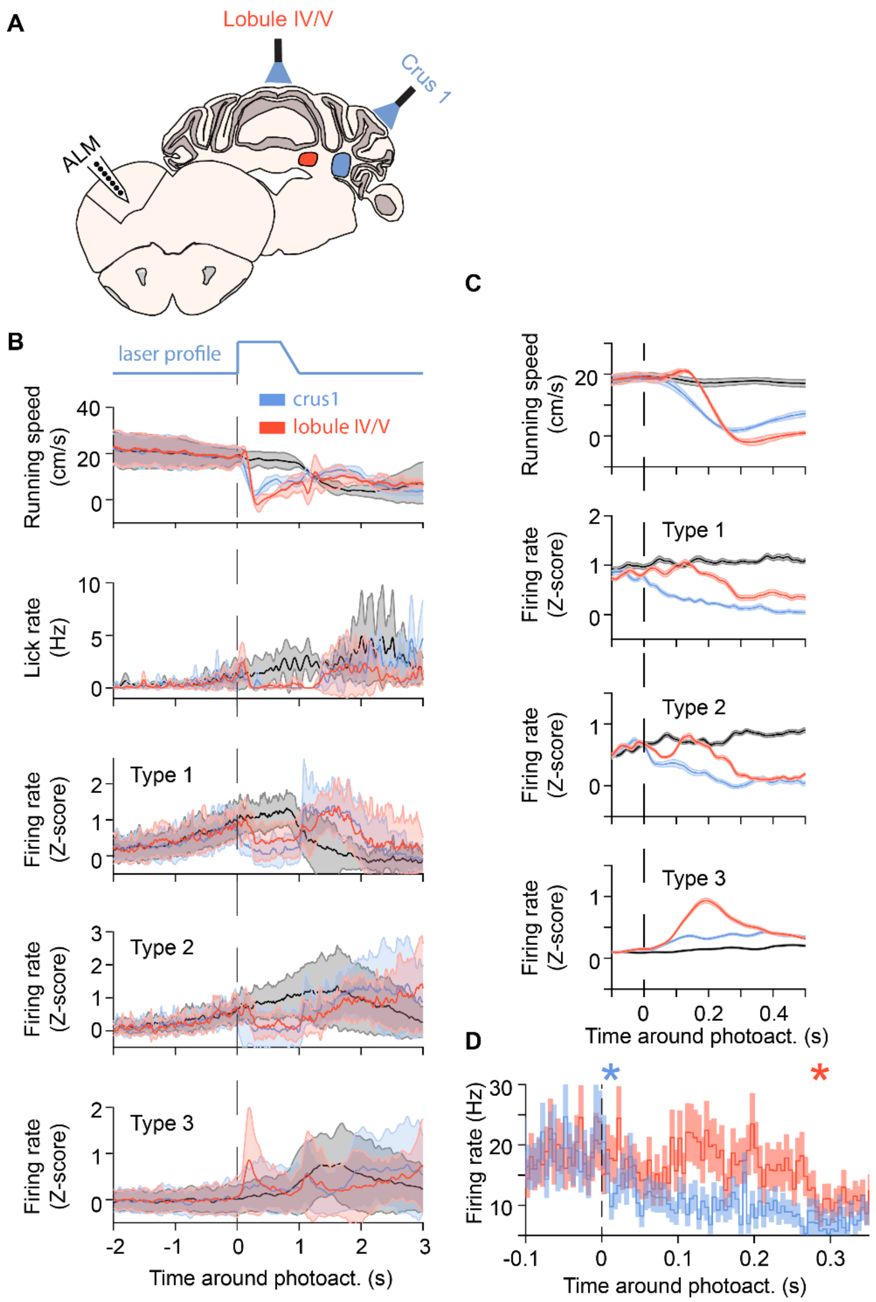

Figure S10. Comparison of the effect of lateral crus 1 and lobule IV-V PC photoactivation on ALM activity (related to Figure 3)

(A) Schematic of experiments. The anterolateral motor cortex (ALM) was recorded in L7-ChR2 mice preforming the task during photoactivation of Purkinje cells (PCs) in lateral crus 1 or lobule IV-V. Lateral crus 1 PCs inhibit the dentate nucleus (blue region) and lobule IV-V PCs inhibit the fastigial nucleus (red region).

(B) From top to bottom, average (line) and standard deviation (shaded area) of running speed, lick rate, and type 1-3 ALM neuronal activity during control (grey), lateral crus 1 (blue), or lobule IV-V photoactivation (red) aligned on photoactivation onset (vertical dotted line). The laser activation profile is illustrated above the running speed traces. A ramping offset was introduced to limit the rebound of neuronal activity.

(C) From top to bottom, average (line) and SEM (shaded area) of running speed, and type 1-3 ALM neuronal activity during the first 500 milliseconds following photoactivation onset.

(D) Average response profiles of firing rate from type 1-2 ALM neurons with significant reduction in activity compared to control trials, during the first 350 milliseconds following PC photoactivation onset (vertical dotted line) in lateral crus 1 (blue) or lobule IV-V (red). Shaded areas represent SEM. Color-coded stars indicate first bin where firing rate significantly deviates from baseline (10 ms for lateral crus 1 photoactivation, 280 ms for lobule IV-V photoactivation; see methods).

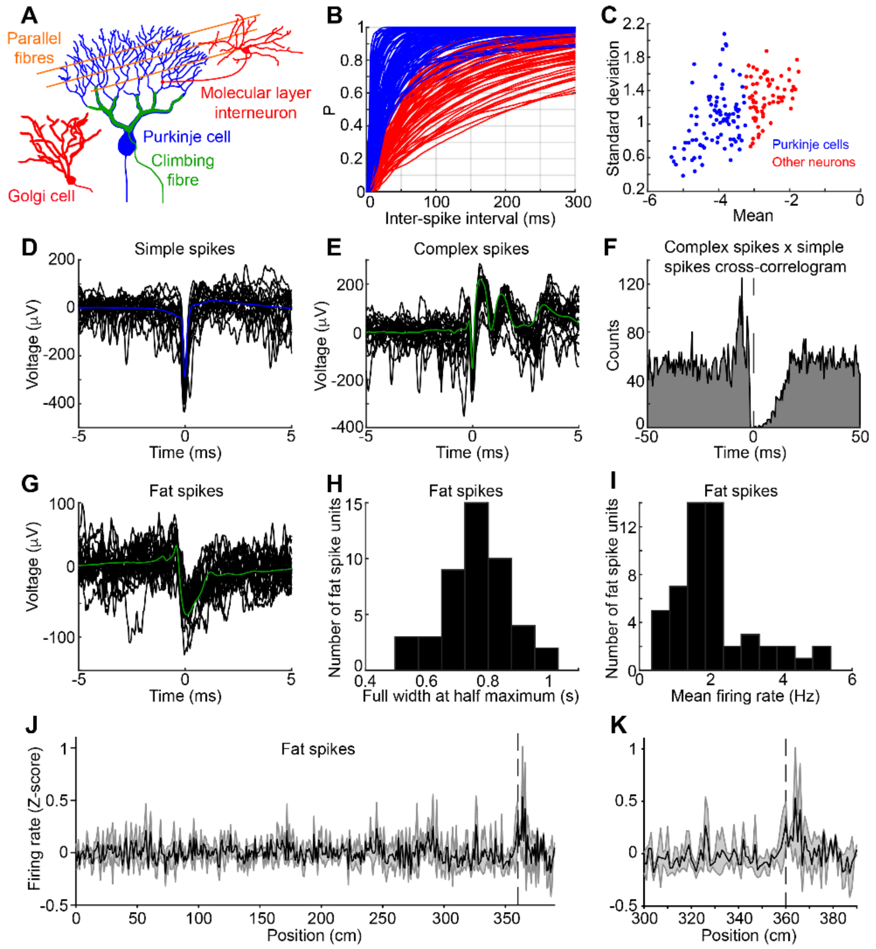

Figure S11. Single unit classification in the cerebellar cortex (related to Figures 4 and 5)

(A) Schematic drawing of cerebellar cortex elements which activity can appear in our extracellular recordings. Purkinje neurons (PCs, blue) are inhibited by molecular layer interneurons (red) and have two sources of inputs: parallel fibres (orange) that give rise to simple spikes and climbing fibre (green) that give rise to complex spikes also recorded as fat spikes. Golgi cells are inhibitory interneurons found in the granule cell layer.

(B) Cumulative distributions of inter-spike intervals (ISI) from units classified as PCs (blue) or other neurons (red). Units shown in this figure where recorded in crus 1 and 2.

(C) ISI distributions were fitted with a log-normal function. K-means clustering with 2 clusters was performed on the mean and standard deviation of the ISI fits to separate putative PCs (blue dots) from other neurons (red dots).

(D) Twenty successive (black) and average (blue) simple spike (SS) waveforms from a putative PC (high-pass filtered at 100 Hz).

(E) Same as (D) for complex spikes (CSs) from the same PC.

(F) Cross-correlogram (0.5 ms binning) between all SSs (examples in D) and CSs (examples in E). Note the characteristic pause in SS firing following the occurrence of a CS and lasting a few tens of milliseconds. All detected CSs belonged to neurons classified as PCs in (C).

(G) Same as (D) for a unit classified as a fat spike (see Methods).

(H) Distribution of fat spikes full width at half maximum. Fat spike duration is significantly longer than axonal spikes and are likely to result from the large depolarisation of PCs dendritic trees induced by climbing fibre discharges.

(I) Distribution of fat spike mean firing rates. Note that those low firing rates are characteristic of climbing fibre activity.

(J) Average firing rate (Z-score) +/- SD of all fat spikes significantly modulated after reward delivery (*n* = 13/26; see Methods). Fat spikes most regularly occur slightly further than the position of reward delivery (360 cm vertical dotted line).
(K) same as (J) focusing on the rewarded section of the corridor.

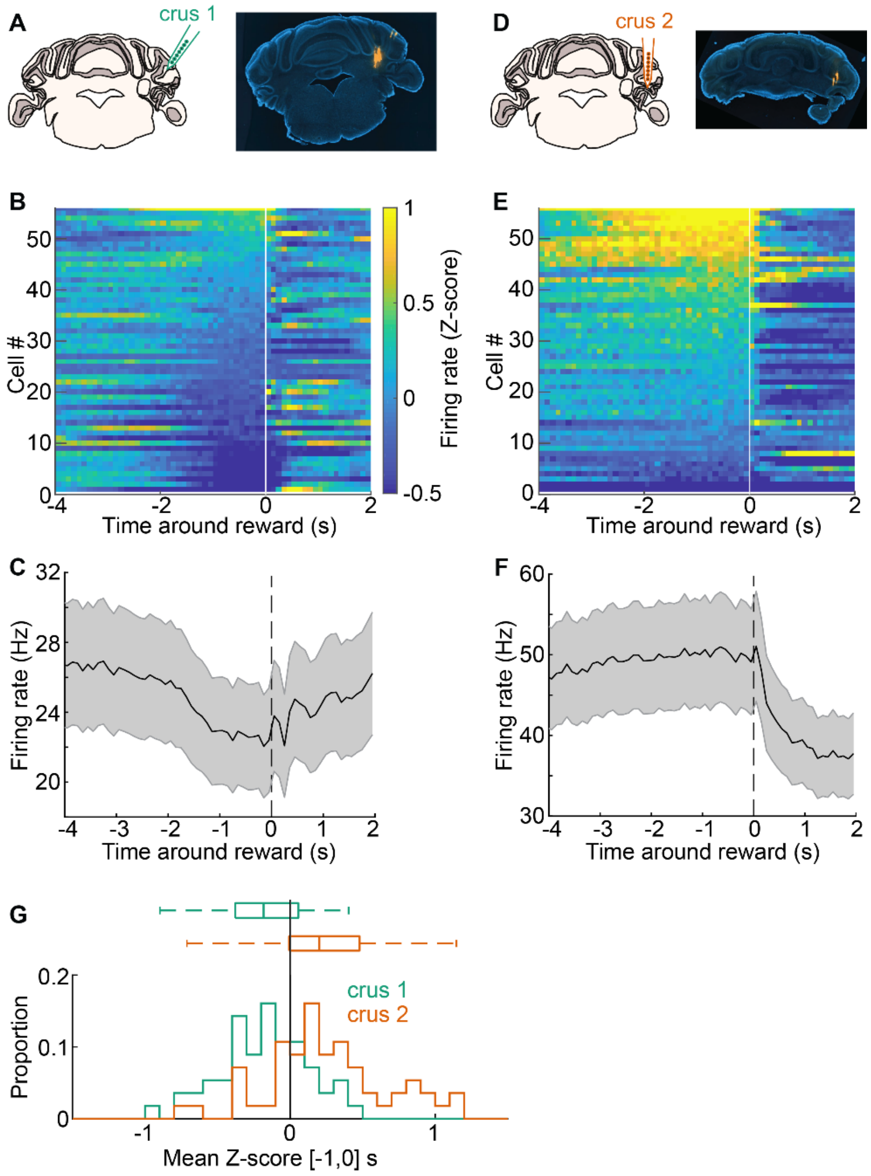

Figure S12. Purkinje cells in crus 2 show little sign of decrease in activity before reward unlike in crus 1 (related to Figure 4)

(A) Left: Schematic of recording location in lateral crus 1. Right: Coronal brain slice from a lateral crus 1 recording (DiI tracks). Not that the DiI tracks in the DCN are from simultaneous DN recording.

(B) Average response profiles for all crus 1 Purkinje cells (PCs) sorted by their mean Z-score value in the last second before reward. White vertical line indicates reward time.

(C) Average PC population firing rate (black line) and SD (grey shaded area) showing decrease of activity starting around two seconds before reward (vertical dotted line).

(D) Same as (A) for recordings in crus 2.

(E) Same as in (B) for crus 2 PCs.

(F) Same as in (C) for crus 2 PCs.

(G) Distribution (bottom) and bar plot (top) of mean Z-score value in the last second before reward for crus 1 (green) and crus 2 PCs (orange; $p < 0.0001$, Wilcoxon Rank-Sum test).
